# Acute kidney injury incidence and mortality following spontaneous intracerebral hemorrhage: a systematic review and meta-analysis

**DOI:** 10.3389/fmed.2026.1721535

**Published:** 2026-03-12

**Authors:** Fuan Zhang, Haomiao Wang, Jiaxuan Li, Rui Liu, Hongxin Zhao, Ping Zhang, Xiangping Xia, Hua Xiao, Chong Han, Shengtao Yao

**Affiliations:** 1 Department of Neurosurgery, Affiliated Hospital of Zunyi Medical University, Zunyi, China; 2 Department of Neurosurgery and Key Laboratory of Neurotrauma, Southwest Hospital, Army Medical University (Third Military Medical University), Chongqing, China; 3 Chengdu Qingyang Maternal and Child Health Hospital, Chengdu, China

**Keywords:** acute kidney injury, cerebral hemorrhage, epidemiology, meta - analysis, systematic review

## Abstract

**Background:**

Intracerebral hemorrhage (ICH) is a neurological emergency that is frequently associated with high mortality and severe disability. Acute kidney injury (AKI) is a common in-hospital complication of ICH, and it is linked to increased mortality risk and poor functional outcomes. However, there have been inconsistent AKI diagnostic criteria across relevant studies in recent years, and there is a lack of updated comprehensive evidence regarding the association between ICH treatment and AKI risk. Therefore, this study aims to systematically investigate the incidence rate of AKI following ICH and its association with mortality in ICH patients.

**Methods:**

We conducted a comprehensive literature search in the Embase, PubMed, and Scopus databases up to April 1, 2025. Two independent reviewers performed data extraction and assessed risk of bias. The Newcastle-Ottawa Scale (NOS) was used for cohort studies, while the Risk of Bias 2 (RoB 2) tool was applied for randomized clinical trials, respectively.

**Results:**

A total of 12 studies, with a total of 16,199 patients, were included in this meta-analysis. The pooled incidence rate of AKI following ICH was 20.4% (95% confidence interval [CI]: 15.2–25.6%). Subgroup analysis based on AKI diagnostic criteria revealed that the incidence rate of AKI diagnosed by KDIGO was higher than that diagnosed by the AKIN. Further analysis of AKI stages showed that stage 1 AKI was the most common type among ICH patients, accounting for a pooled proportion of 75.6% (95% CI: 59.9–91.4%), while stages 2 and 3 AKI had similar proportions. Additionally, AKI was linked to a higher short-term mortality risk in ICH patients, with a pooled odds ratio (OR) of 3.16 (95% CI: 1.93–5.18).

**Conclusion:**

AKI is common in ICH and associated with higher short-term mortality. Future studies should focus on early AKI biomarkers, individualized blood pressure control and long-term prognosis.

**Systematic review registration:**

CRD42025642481.

## Introduction

Currently, intracerebral hemorrhage (ICH) ranks among the most devastating neurological conditions worldwide, standing as a leading driver of global mortality and disability-adjusted life years (DALYs) (1–3), imposing a heavy burden on both affected patients and society at large. Against this backdrop, acute kidney injury (AKI), a common in-hospital complication in ICH cases, has become an increasing clinical concern due to its association with higher short- and long-term mortality, poor functional outcomes, and its rising incidence (4–6).

The conceptualization of this renal complication has evolved significantly. Initially termed acute renal failure (ARF), this designation was plagued by inconsistent definitions and a failure to capture early pathophysiological changes in renal damage. In response, the Acute Dialysis Quality Initiative Group (ADQI) proposed the Risk, Injury, Failure, Loss of kidney function, and End-stage kidney disease (RIFLE) criteria for the diagnosis and staging of AKI (7), the first standardized criteria for AKI staging that divides renal injury into 5 hierarchical stages based on serum creatinine (Scr) changes and urine output reduction, which addressed the lack of uniform ARF definitions and enabled the first stratification of renal injury severity in clinical practice. The Acute Kidney Injury Network (AKIN) subsequently revised these criteria to establish the AKIN standards (8), simplifying RIFLE into 3 stages, narrowing the Scr change time window to 48 h and lowering the Scr elevation threshold to 1.5 times the baseline, a revision designed to facilitate early clinical identification of AKI and simplify staging for rapid bedside application. In 2012, the Kidney Disease: Improving Global Outcomes (KDIGO) initiative issued updated guidelines (9) that built on RIFLE and AKIN to address the high misdiagnosis rates of prior classification systems; this criterion retained the 3-stage classification, expanded the Scr and urine output judgment thresholds and incorporated definitions of persistent renal injury, which improved the detection of mild and early AKI, featured more comprehensive staging suitable for critically ill patient populations such as post-ICH patients, and standardized AKI diagnosis across different studies. This evolutionary process, however, has led to inconsistent AKI definitions across post-ICH studies, hindering direct comparisons of their findings (4), with the core differences among the three criteria lying in that RIFLE focuses on the stratification assessment of moderate to severe renal injury, AKIN prioritizes rapid early diagnosis with a narrow time window for Scr changes, and KDIGO balances early AKI detection and severity stratification with the most sensitive diagnostic threshold for early renal injury.

Besides, therapeutic approaches for ICH have expanded rapidly. Large multicenter randomized trials and guidelines from the American Heart Association/American Stroke Association indicate that timely, intensive acute reduction of systolic blood pressure (SBP), an intervention involving the prompt titration of SBP to guideline-recommended target levels (<140 mmHg) in patients with acute ICH, is safe and improves functional outcomes in ICH patients (10–13). However, findings from ACTH-1/2 trials (14, 15) and other observational studies (6, 16) suggest intensive SBP reduction may correlate with renal impairment and AKI. Additionally, life-saving interventions like mannitol, which have been widely used in clinical practice for ICH, have been associated with AKI in these patients (17). Given these conflicting links between primary ICH therapies and AKI risk, a clearer understanding of post-ICH AKI has become critically urgent.

To our knowledge, although there have been several important research updates in this field, no updated systematic reviews or meta-analyses have further explored and summarized this critical clinical issue. Given the significance, we conducted this systematic review and meta-analysis to investigate the incidence and mortality of AKI following ICH and evaluate the impact of post-ICH AKI on patients’ risk of death.

## Methods

### Data sources

This systematic review and meta-analysis followed the PRISMA (Preferred Reporting Items for Systematic Reviews and Meta-Analyses) guidelines. The study protocol was registered in PROSPERO with the registration number CRD42025642481. A comprehensive literature search was performed in the Embase, PubMed, and Scopus databases up to April 1, 2025, with no restrictions on data.

### Eligibility criteria and data extraction

Studies meeting the inclusion criteria were divided into two categories: those describing the incidence rate of acute kidney injury (AKI) after intracerebral hemorrhage (ICH), and those evaluating any association between AKI and mortality in ICH patients. We included studies involving adult patients with ICH confirmed by CT or MRI, and excluded those including traumatic ICH or subarachnoid hemorrhage. Similarly, we excluded studies that evaluated the incidence of contrast-induced or mannitol-induced kidney injury in ICH patients. In this present study, two studies used data from the same patient population but applied different AKI diagnostic criteria; thus, they were identified as separate studies and included in the analysis (18, 19).

Two authors (F.Z., a professional neurosurgeon, and J.L., a professional nurse with neurosurgical nursing experience) first independently screened titles and abstracts and subsequently evaluated the full texts to determine whether the studies met the predefined inclusion criteria. Any discrepancies arising during the screening process were resolved through discussion, with the senior author (S.Y., an experienced neurosurgeon) involved in making final decisions. Additionally, these two authors first pretested the data extraction form before independently extracting outcome data. They then cross-checked their extracted results, and any inconsistencies were resolved through discussion to reach a consensus.

### Outcomes

The primary and secondary outcomes included in the analysis were the incidence rate of AKI and all-cause mortality. In this study, AKI was defined based on three diagnostic criteria: Risk, Injury, Failure, Loss of kidney function, End-stage kidney disease (RIFLE), Acute Kidney Injury Network (AKIN), and Kidney Disease: Improving Global Outcomes (KDIGO). This definition was determined by assessing kidney function using serum creatinine and the Modification of Diet in Renal Disease (MDRD) formula to calculate estimated glomerular filtration rate (eGFR) (8).

### Assessment of risk of bias

Two authors (R.L. and H.Z.) independently assessed the risk of bias using the Newcastle-Ottawa Scale (NOS) for cohort studies and the Risk of Bias 2 (RoB 2) tool for randomized clinical trials and studies with a NOS score of ≥7 were classified as low-bias studies (20, 21). Any disagreements that arose during this process were resolved through consensus.

### Statistical analysis

The pooled odds ratios (ORs) for the association between AKI and mortality were converted using natural logarithms, and standard errors (SE) were calculated from their respective 95% confidence intervals (CIs). No covariates were incorporated into the model for adjustment. Furthermore, we conducted a sensitivity analysis, excluding ORs that derived from study that ICH patients receiving intensive blood pressure reduction. For heterogeneity assessment, we reported Cochran’s Q statistic, *I*^2^ value, and *τ*^2^ statistic, and categorized *I*^2^ values: *I*^2^ < 25% was defined as low heterogeneity, 25–50% as moderate heterogeneity, and >50% as high heterogeneity. All analyses were performed using R software.

## Results

### Overview of included studies

**Figure 1:**
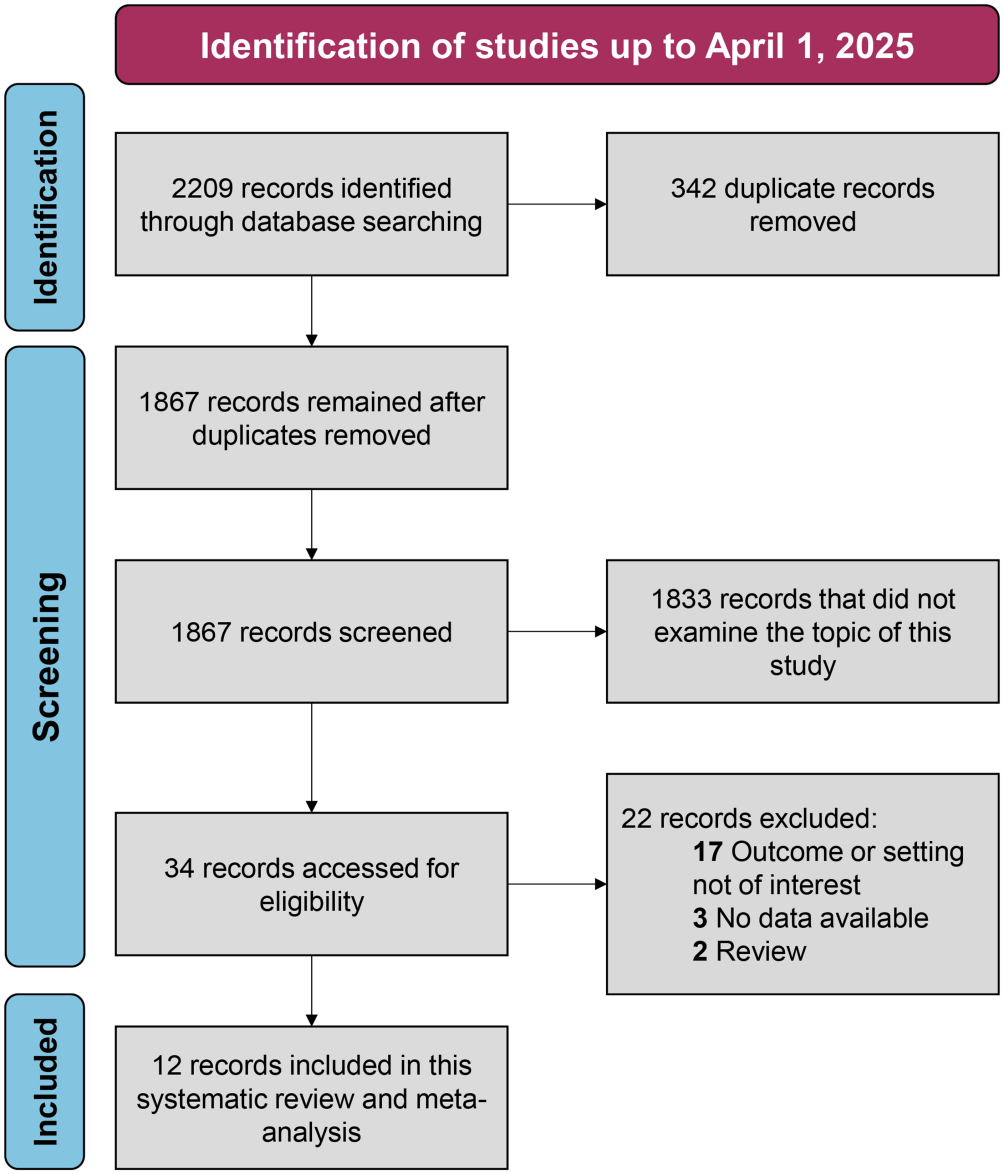
The flow chart for the selection of studies in this systematic review and meta-analysis.

We conducted searches and identified 2,209 records. Following the removal of duplicates, 1867 unique records remained. Subsequent screening of titles and abstracts led to the exclusion of 1833 records as they did not meet the inclusion criteria. We assessed the full texts of the remaining 34 records, and finally, we included 12 studies (6, 14, 18, 19, 22–29) in the meta-analysis and systematic review. The detailed flow chart is presented in Figure 1.

Overall, the 12 studies included in the meta-analysis comprised 16,199 patients, with 5 (41.7%) being prospective studies and 7 (58.3%) being retrospective studies. For defining post-ICH AKI, 7 (58.3%) of these studies used the Kidney Disease: Improving Global Outcomes (KDIGO) criteria, 4 (33.3%) used Acute Kidney Injury Network (AKIN), and 1 (8.3%) used Risk, Injury, Failure, Loss of kidney function, End-stage kidney disease (RIFLE). Notably, two of these studies (18, 19), both with data from the ACTH-2 trial, used AKIN and KDIGO, respectively, to identify and define AKI following ICH. The baseline characteristics of the included studies are presented in Table 1.

**Table 1:** Overview and characteristics of studies included in the meta-analysis.

Study	Age	AKI criteria	AKI, *n* (%)	AKI mortality, *n* (%)	Non-AKI mortality, *n* (%)
Tian 2023	58.1	KDIGO	1,325 (13.7)	NA	NA
Wang 2023	69.8	KDIGO	356 (29.3)	132 (37.0)	236 (27.5)
Naidech 2023	59.5	KDIGO	109 (10.9)	NA	NA
Zhang 2021	58.6	KDIGO	136 (9.4)	70 (51.5)	299 (22.8)
Wang 2020	58.0	KDIGO	142 (35.2)	NA	NA
Qureshi 2020	59.5	AKIN	149 (14.9)	21 (14.1)	46 (5.4)
Ansaritoroghi 2019	56.3	KDIGO	64 (20.3)	10 (15.6)	14 (5.5)
Jiang 2019	NA	KDIGO	90 (29.8)	NA	NA
Burgess 2018	62.0	AKIN	139 (31.0)	55 (39.6)	37 (12.0)
Khatri 2014	64.7	AKIN	171 (20.6)	68 (39.8)	200 (30.4)
Qureshi 2012	62.0	AKIN	5 (8.3)	2 (40.0)	8 (14.5)
Covic 2008	66.1	RIFLE	35 (22.2)	NA	NA

AKI, acute kidney injury.

### Quality assessment

Cohort studies and randomized clinical trials were assessed for quality using the Newcastle-Ottawa Scale (NOS) and the Risk of Bias 2 (RoB 2) tool, respectively. All included studies were judged to have a low risk of bias (Supplementary Figure S1 and Supplementary Table S1).

### Incidence of AKI following ICH

**Figure 2:**
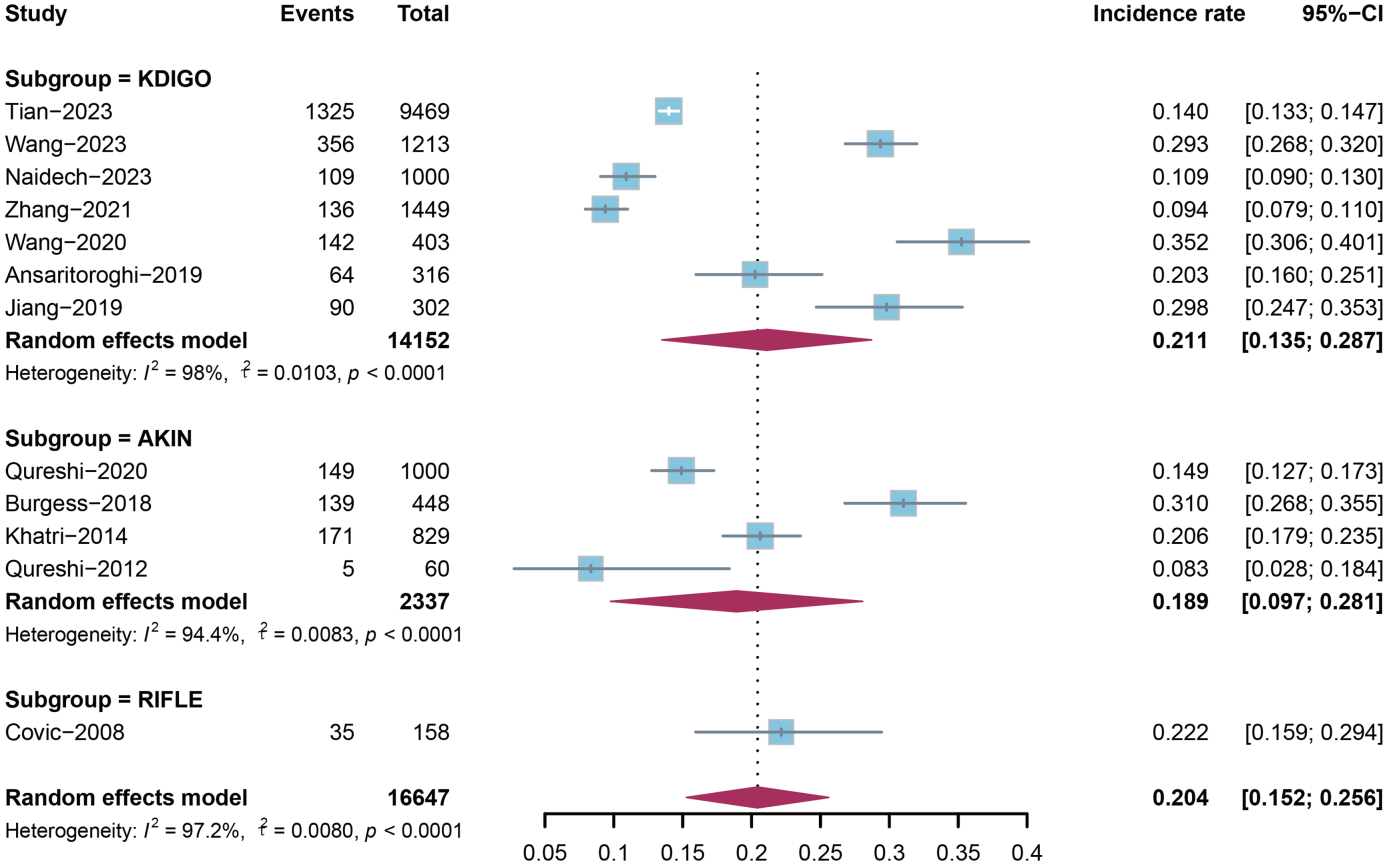
The forest plot showing pooled incidence rate of AKI after ICH. AKI, acute kidney injury; ICH, intracerebral hemorrhage.

As shown in Figure 2, the summary incidence rate of acute kidney injury (AKI) following intracerebral hemorrhage (ICH) is 20.4% (95% confidence interval [CI]: 15.2–25.6%), with statistically significant heterogeneity (*I*^2^ = 97.2%, *p* < 0.0001). Further, we conducted subgroup analyses based on post-ICH AKI definitions, including KDIGO, AKIN, and RIFLE. The pooled incidence of AKI in ICH patients diagnosed by KDIGO was 21.1% (95% CI: 13.5–28.7%), while that diagnosed by AKIN was 18.9% (95% CI: 9.7–28.1%). Only one study used the RIFLE criteria, which reported an AKI incidence of 22.2% (95% CI: 15.9–29.4%) in ICH patients.

Subgroup analyses were conducted to compare the incidence rates across the three diagnostic criteria, and statistically significant heterogeneity was noted in each subgroup. Meanwhile, the incidence rates determined by the KDIGO and RIFLE criteria were slightly higher than that by the AKIN criteria, a finding consistent with previous reports (30, 31) that the KDIGO criteria has the highest diagnostic rate of AKI in hospitalized patients, the RIFLE criteria is comparable to it, and both are superior to the AKIN criteria.

Given that the stage of AKI in ICH patients is associated with prognosis, we further analyzed the proportions of different post-ICH AKI stages (Supplementary Figures S2–S4). A total of 5 studies staged post-ICH AKI with 2 studies using the KDIGO criteria and 3 using AKIN. Post-ICH AKI stage 1 is the most common type with a pooled incidence rate of 75.6% (95% CI: 59.9–91.4%). The proportions of AKI stage 2 and stage 3 are similar. For AKI stage 3, incidence varied widely across studies, ranging from 0 to 40.4%. The analysis showed statistically significant heterogeneity.

### Mortality of AKI following ICH

**Figure 3:**
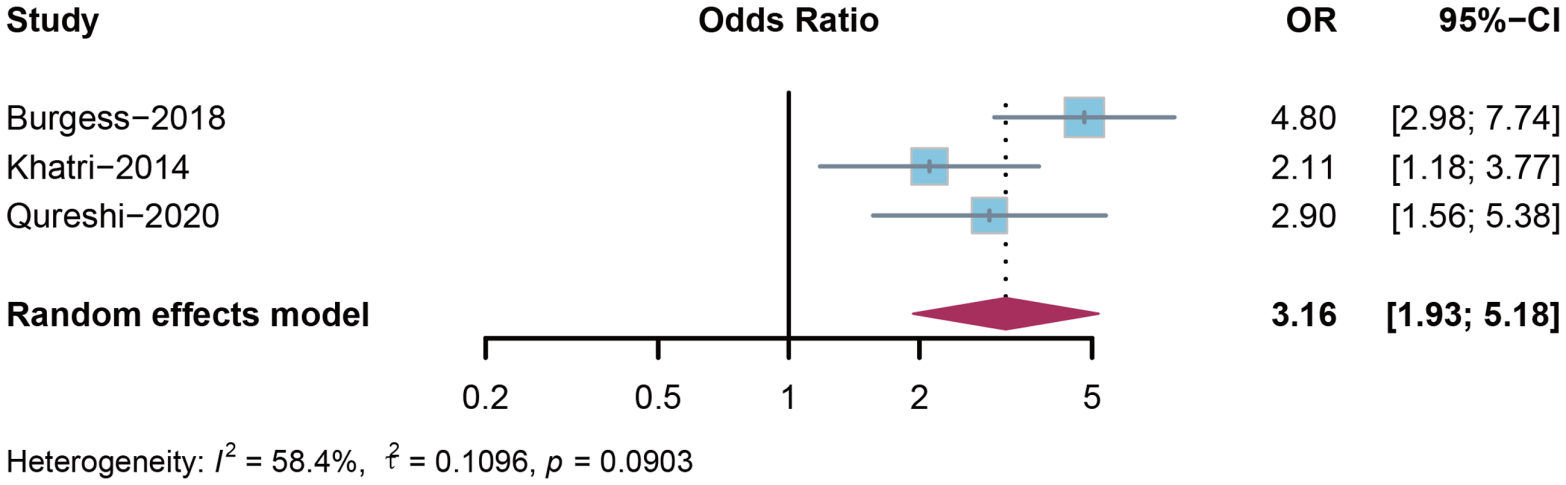
The forest plot showing the adjusted OR for the association between AKI and mortality in patients with ICH. OR, Odds ratio; AKI, acute kidney injury; ICH, intracerebral hemorrhage.

AKI is recognized as a risk factor for both short- and long-term mortality in ICH patients. Thus, we aimed to assess the mortality risk in ICH patients who experienced AKI. A total of 3 studies were included in the analysis with 2 with in-hospital mortality, and 1 with 90-day follow-up. Shown in Figure 3, the results from the meta-analysis showed a significantly increased mortality risk (odds ratio [OR] 3.16; 95% CI: 1.93–5.18; *I*^2^ = 58.4%; *p* = 0.0903).

### Sensitivity analysis

Considering that the occurrence of AKI in patients with ICH has been reported to be associated with intensive blood pressure reduction, we performed additional analysis on the mortality risk related to AKI in these patients. Specifically, we included studies that investigated mortality outcomes in ICH patients who underwent intensive blood pressure reduction and developed AKI. Ultimately, 2 studies were included in the analysis. Consistent with the previous analysis, similar results were observed (Supplementary Figure S5) that ICH patients with post-ICH AKI had a significantly increased risk of mortality compared to those without AKI (OR 3.88; 95% CI: 2.38–6.32; *I*^2^ = 37.5%; *p* = 0.2058). Due to the fact that the number of studies included in the meta-analysis is still comparatively small, a factor that undermines the validity of funnel plots in detecting publication bias, we chose not to conduct funnel plot to assess the publication bias.

## Discussion

This systematic review and meta-analysis included 12 studies with 16,647 ICH patients. We found that the pooled incidence of AKI after ICH onset was 20.4% (95% CI: 15.2–25.6%), which is clinically significant. Across the studies, heterogeneity was high for the incidence of post-ICH AKI. Subgroup analysis based on different diagnostic criteria for AKI showed distinct differences in the incidence of AKI after ICH. Compared with the KDIGO criteria, the incidence of AKI after ICH diagnosed by the AKIN criteria was lower. This finding may align with previous studies, which confirmed that the KDIGO criteria are more sensitive and accurate in identifying early kidney injury than the AKIN criteria (31). These results suggest that the incidence of AKI after ICH might have been underestimated in some previous studies that used the AKIN criteria. However, two separate studies from the ACTH-2 trial, which applied the AKIN and KDIGO criteria respectively, revealed the opposite trend (18, 19). Specifically, the incidence of new-onset AKI after ICH under the AKIN criteria was 15%, which was higher than the 11% observed under the KDIGO criteria. In addition, significant heterogeneity was noted across all included studies. Therefore, more studies are needed in the future to further explore and compare the applicability of different AKI diagnostic criteria, with the goal of establishing the most suitable standard for identifying AKI after ICH.

For patients with ICH, most require contrast-based diagnostic imaging to confirm the diagnosis, while some also receive mannitol therapy for cerebral edema, and both of these practices increase the risk of AKI (17, 32). Additionally, as ICH is a neurological emergency, most patients need surgical intervention or admission to the intensive care unit for monitoring and treatment. During this period, they may be given nephrotoxic antibiotics such as vancomycin and fluoroquinolones, which further raises the risk of kidney damage (25).

Notably, ICH patients with severe hypertension are more likely to develop AKI, as reported in previous studies (16). To reduce the risk of AKI, it is necessary to maintain relatively high blood pressure levels in these patients (6, 14, 18), and this creates a certain contradiction with the currently used intensive blood pressure reduction, which is a treatment widely recognized to improve mortality and functional outcomes in ICH patients (33). In this study, we found ICH patients who develop AKI have a short-term mortality rate of 14.1–43.1%, which is significantly higher than that of ICH patients without AKI. The pooled OR for short-term mortality risk in these ICH patients with AKI was 3.16 (95% CI: 1.93–5.18). When only the study that ICH patients receiving intensive blood pressure reduction was included and a sensitivity analysis was conducted, the OR increased to 3.88 (95% CI: 2.38–6.32), suggesting the efficacy of intensive blood pressure reduction should be further tested. Besides, a proportion of ICH patients already have chronic kidney disease (CKD) before the onset of ICH. CKD is not only a risk factor for ICH but also increases the likelihood of AKI in patients after ICH (25, 34), and applying intensive blood pressure reduction to this population further exacerbates this risk (35). Going forward, further studies are needed to determine the optimal blood pressure reduction strategy for these ICH patients.

A national study (36) reported that between 2002 and 2011, the incidence of AKI requiring dialysis among ICH patients showed an upward trend. During the same period, the short-term mortality rate and adverse discharge outcomes also rose significantly in ICH patients who needed dialysis. Among ICH patients with AKI, there are notable differences in renal function recovery across different AKI stages. Patients with AKI Stage 1 or 2 have a significantly higher renal function recovery rate than those with Stage 3. Specifically, AKI Stage 3 patients are more likely to progress to renal failure, and some of them require dialysis (37). Furthermore, the mortality rate of these patients who need renal replacement therapy is significantly higher during long-term follow-up (25). These results suggest that for the vulnerable group of patients with both ICH and AKI, it is important to carry out early diagnosis, optimize risk stratification, and make preparations for their complex long-term care needs.

In recent years, significant progress has been made in biomarker research for AKI following ICH. β2-microglobulin has been identified as an independent predictor of AKI in ICH patients, with its expression levels closely associated with in-hospital and long-term mortality (22). The lactic dehydrogenase-to-albumin ratio serves as a cost-effective and reliable biomarker for predicting AKI risk after ICH, demonstrating important clinical value in early identification and risk stratification (38). Additionally, procalcitonin has been shown to predict AKI occurrence and the need for renal replacement therapy in ICH patients (39). However, novel biomarkers such as NGAL and KIM-1 await specific validation in ICH populations, and future research should focus on identifying optimal biomarker combinations to establish an early warning system. Regarding predictive models, the ICH-AKIM score integrates five clinical variables (sex, systolic blood pressure, diabetes history, Glasgow Coma Scale score, and mannitol use) and four laboratory parameters (serum creatinine, albumin, uric acid, and neutrophil-to-lymphocyte ratio), exhibiting excellent predictive performance (24). With advances in artificial intelligence technologies such as machine learning, more precise predictive tools for AKI after ICH are anticipated to support clinical decision-making.

Several limitations of this study should be acknowledged. First, in the meta-analysis of AKI incidence among ICH patients, significant high heterogeneity was observed, which may limit the generalizability of our conclusions. To address this, we used selected random-effects model and performed subgroup analyses stratified by different AKI diagnostic criteria to obtain a more accurate incidence rate estimate. Second, most studies included in this analysis are single-center cohort studies, which may introduce recall bias and therefore requires cautious interpretation of the results. Third, preexisting CKD and other confounders, including age, SBP and treatment, are known risk factors for AKI in ICH patients and are also associated with mortality and functional outcomes. This makes it challenging to establish a causal relationship between AKI and mortality in ICH patients. Nevertheless, AKI still emerges as a potential risk factor of death and poor functional outcome that warrants attention in clinical practice. Additionally, only short-term mortality was analyzed due to the lack of data on long-term mortality and functional outcomes, highlighting the need for future studies to explore these outcomes.

## Conclusion

Through our systematic review and meta-analysis, AKI is confirmed as a common post-ICH complication (19% incidence rate), and it is associated with a notably higher mortality risk as well as unfavorable functional outcomes in affected ICH patients. Future studies should be conducted focusing on identifying reliable biomarkers for early AKI detection in ICH patients, exploring individualized blood pressure control plans to balance ICH outcomes and AKI risk, analyzing long-term impacts of AKI on the renal function of ICH patients, and standardizing nursing care pathways for AKI management. In addition, more studies should assess the safety and effectiveness of intensive blood pressure reduction in ICH patients with preexisting CKD.

## Data Availability

The original contributions presented in the study are included in the article/Supplementary material, further inquiries can be directed to the corresponding authors.
