# Supplementary material for: Acute kidney injury incidence and mortality following spontaneous intracerebral hemorrhage: a systematic review and meta-analysis
Source: Front Med (Lausanne). 2026 Mar 12;13:1721535. doi: 10.3389/fmed.2026.1721535 (PMC13018104; doi:10.3389/fmed.2026.1721535)
Supplement: Supplementary file 1 [file Data_Sheet_1.pdf]

Table S1. Risk of bias assessment of included cohort studies by the Newcastle-Ottawa Scale.

	**Selection**				**Comparability**	**Outcome**			**Total score**
**First author, published year**	**Representativeness of the Exposed Cohort**	**Selection of the Non-Exposed Cohort**	**Ascertainment of Exposure**	**Demonstration That Outcome Of Interest Was Not Present at Start of Study**	**Comparability of Cohorts on the Basis of the Design or Analysis**	**Assessment of Outcome**	**Was Follow-Up Long Enough for Outcomes to Occur**	**Adequacy of Follow Up of Cohorts**	**Risk of bias**
Tian-2023	1	1	1	1	2	1	1	1	9
Wang-2023	1	1	1	1	1	1	1	1	9
Zhang-2021	1	1	1	1	2	1	1	1	9
Wang-2020	1	1	1	1	2	1	1	1	9
Ansaritoroghi-2019	1	1	1	1	2	1	1	1	9
Jiang-2019	1	1	1	1	2	1	1	1	9
Burgess-2018	1	1	1	1	2	1	1	1	9
Khatri-2014	1	1	1	1	2	1	1	1	9
Covic-2008	1	1	1	0	1	1	1	1	8

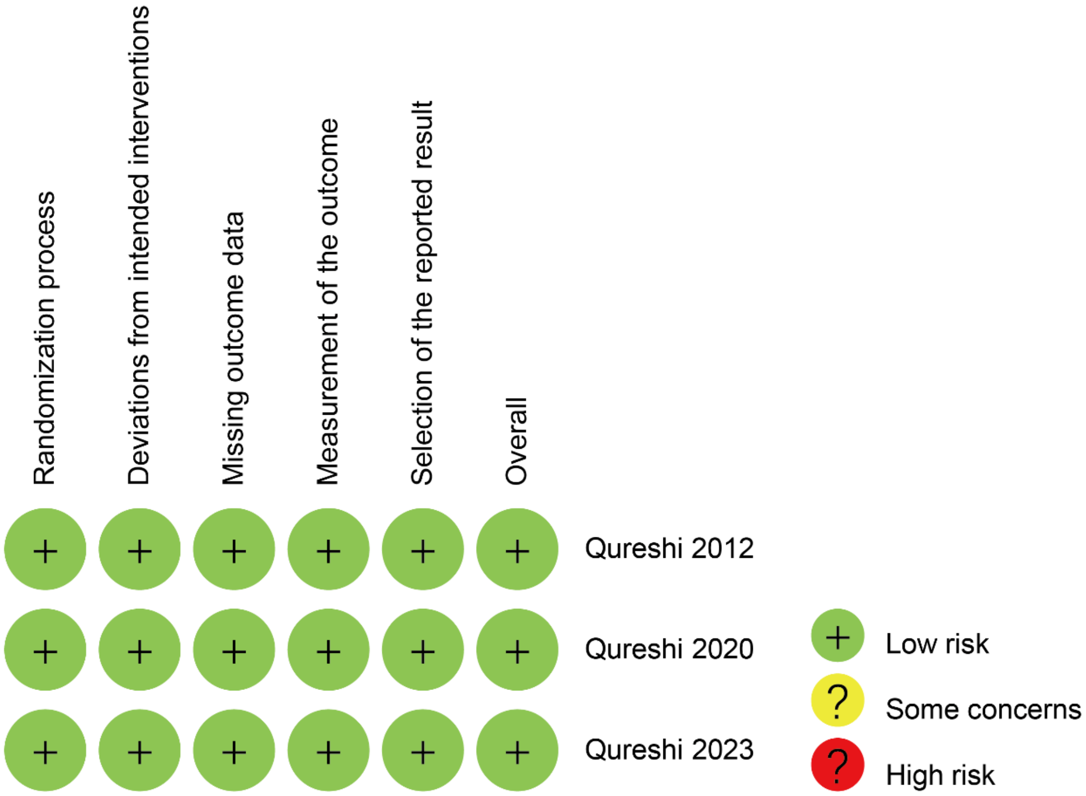

Figure S1. Risk of bias assessment of included randomized clinical trials by Risk of Bias 2 tool.

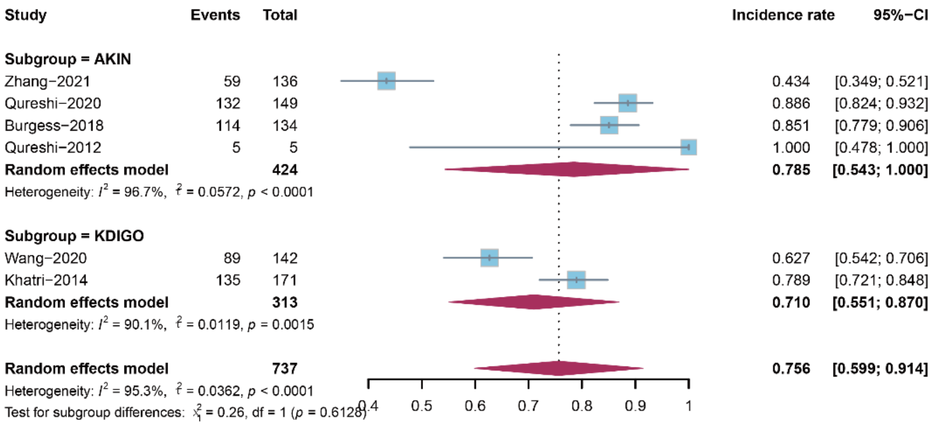

Figure S2. The forest plot showing pooled proportion of stage 1 AKI after ICH. AKI, acute kidney injury; ICH, intracerebral hemorrhage.

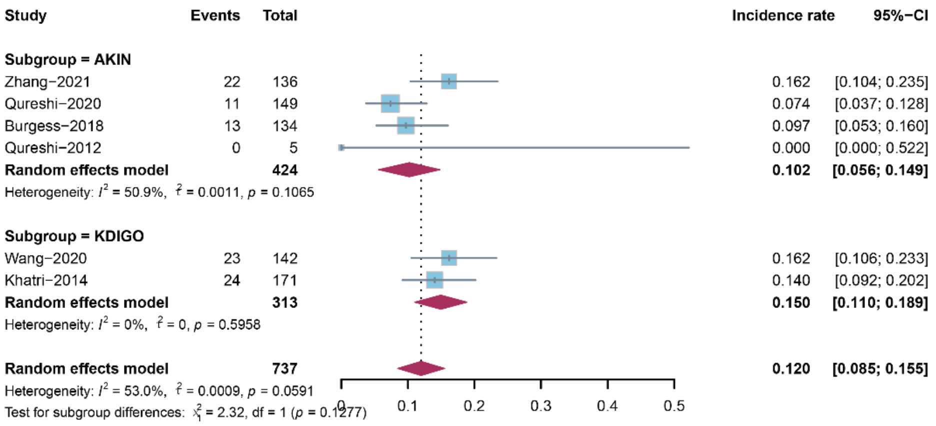

Figure S3. The forest plot showing pooled proportion of stage 2 AKI after ICH. AKI, acute kidney injury; ICH, intracerebral hemorrhage.

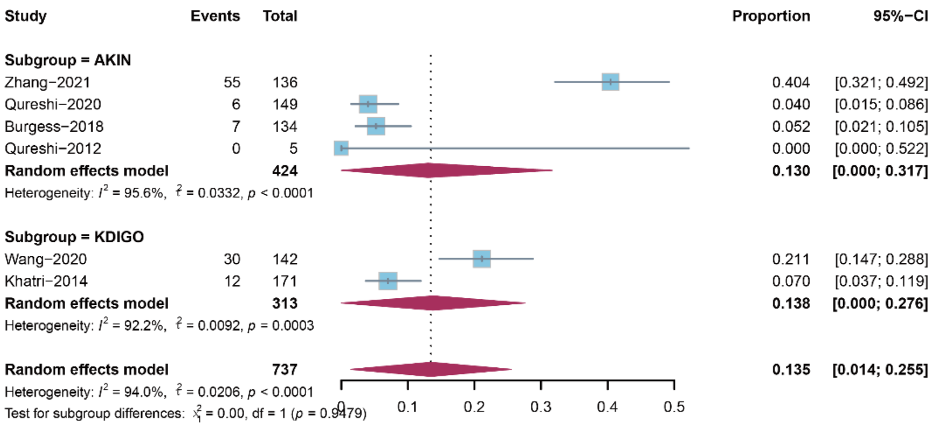

Figure S4. The forest plot showing pooled proportion of stage 3 AKI after ICH. AKI, acute kidney injury; ICH, intracerebral hemorrhage.

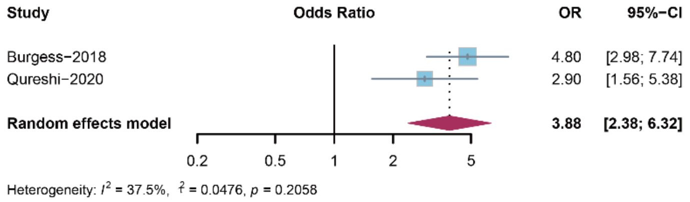

Figure S5. The forest plot of sensitivity analysis showing the adjusted OR for the association between AKI and mortality in patients with ICH. OR, odds ratio; AKI, acute kidney injury; ICH, intracerebral hemorrhage.
